# An Address to the Students of Medicine of the University of Pennsylvania

**Published:** 1818

**Authors:** James Carver

**Affiliations:** V. S.


### ADDRESS

TO THE

### STUDENTS OF MEDICINE,

OF THE

### UNIVERSITY OF PENNSYLVANIA.

---

GENTLEMEN,

It is now fifteen years since I first commenced the arduous undertaking of endeavouring to lay the foundation stone for the better introduction and dissemination of the practice of the Veterinary Art in this city; but owing to the death of our late and much to be lamented friend and patron of medicine, Dr. Benjamin Rush, together with a variety of other causes, I have not been able to fulfil the wishes and desires of a mind naturally bent for these pursuits. I have, however, in some former publications, endeavoured to draw your attention to the subject now before you, with an intention of opening a school of instruction to a course of private lectures on the anatomy, physiology, and pathology of the horse and other quadrupeds, by which means this useful and necessary branch of science would gradually have became dispersed throughout our union, and whereby you would have been rendering yourselves useful in your respective neighbourhoods, by receiving instruction on those heads, and thereby learning how to perform a few useful operations, as well as the administering of medicines where no regular or scientific aid could be procured.

Sensible of the advantages that might have been made to result by obtaining a small share of knowledge in comparative anatomy, you would have been enabled in this way without disparagement to your own profession, to have rendered a useful piece of service to yourselves as well as your country; but from circumstances unavoidable, my pursuits are now turned into a different channel, and I shall thereby endeavour to fulfil this task myself, by travelling through the country. If, however these pages should fall under your eye, let me entreat that you follow me attentively through them, and not be deterred from investigating them; by which, perhaps, each succeeding day of study will open to your view, a new intellectual ray of light, in researches perhaps hitherto unknown to you, and each new fact will forcibly impress itself on your minds—until that which was begun with a dread of being styled a horse doctor, will end in pleasure: for it is by a due and proper acquirement of some of these important branches of new medical information, that you may hope to build some reputation in veterinary knowledge, as a guide in your future professional pursuits.

For more than five and twenty years I have studied the veterinary profession, though not scientifically until I entered on the pursuit at the Veterinary College of London, where, by strict attention, I flatter myself I acquired some knowledge of it. I therefore, from the best of motives, invite your attention to the pages before you, under a hope that the information contained therein may, to some of you, at least prove a pleasing task.

In the descriptive detail of the different diseases, and their modes of cure, I have carefully avoided theory alone, and endeavoured to join cause and effect, and blend the reason with the act; whereby, attempting to teach the curative art by principles more than by recipes, and the matter so conducted, that the amateur who chooses to strip it of its systematic and artificial dress, may find in the work a ready and safe guide which will not lead him astray; and though I have borrowed some matter, that which is my own will stand in no fear of contradiction.

I am, gentlemen,

Very respectfully,

Your obedient servant,

J. CARVER, *V. S.*

